# Preregistration of exploratory research: Learning from the golden age of discovery

**DOI:** 10.1371/journal.pbio.3000690

**Published:** 2020-03-26

**Authors:** Ulrich Dirnagl

**Affiliations:** QUEST Center for Transforming Biomedical Research, Berlin Institute of Health, Berlin, Germany

## Abstract

Preregistration of study protocols and, in particular, Registered Reports are novel publishing formats that are currently gaining substantial traction. Besides rating the research question and soundness of methodology over outstanding significance of the results, they can help with antagonizing inadequate statistical power, selective reporting of results, undisclosed analytic flexibility, as well as publication bias. Preregistration works well when a clear hypothesis, primary outcome, and mode of analysis can be formulated. But is it also applicable and useful in discovery research, which develops theories and hypotheses, measurement techniques, and generates evidence that justifies further research? I will argue that only slight modifications are needed to harness the potential of preregistration and make exploratory research more trustworthy and useful.

## Abstract 2

Pre-registration and registered reports of research that develops theories and hypotheses has the potential to reduce waste and increase the value of biomedical research. This Perspective argues that journals and scientists should develop and experiment with pre-registration formats for discovery research.

## Academic publishing in transition

After decades of relative stability, academic publishing is experiencing a dramatic transition, upending publishers’ business models and also key elements of the publication process, such as how articles are structured, the nature of their content, and how—or even if—they are reviewed. This transformation is triggered by a number of factors: research outputs often lack transparency and rigor and are poorly reproducible, and the research community is overwhelming itself with its own outputs. Which of these published findings are trustworthy, relevant, and useful? In addition, researchers and funders are questioning the justification of publishers’ disproportionate profits. Technical advances and the internet have facilitated the formatting and communication of research results, raising the question of whether we need publishers at all [1].

Interestingly, besides a few activists, scientists are not the drivers of this transformation. They seem too busy with their research—and are caught in a system in which funding and tenure are closely tied to an individual’s publication record. In fact, the academic incentive system is the strongest remaining bulwark protecting the status quo of the publishing industry and its hierarchies of journals. Funding decisions and professors are made by benchmarking top tier publications. Instead, funders, learned societies, and even publishers themselves are transforming the way we disseminate our results. Motivated by the current concerns about the robustness and value of research, they are keen to leverage novel publishing formats to increase research quality, reporting, and dissemination.

Some elements of the novel publishing formats are already conceptually resolved, and their implementation is under way. Barrier-free access (“Open Access”) to all publicly funded research outputs is mandated by many funders, including the European Union. Self-publication of articles before formal review, so-called preprints [2], are already a mainstay of scientific communication in many fields (e.g., mathematics, physics) and are being rapidly adopted in biomedical research (Biorxiv, Medrxiv). About one third of preprints posted at Biorxiv never get published in a peer-reviewed journal [3].

## Peer review, quo vadis?

A number of other novel approaches in academic publishing are less well developed and need more discussion and experimentation as well as implementation research. Many of them revolve around the peer review process, which, for more than half a century, has served as the foundation of quality control in publishing. Clandestinely conducting a study and subsequently presenting its results to peers, followed by an equally clandestine editorial decision, has thus far been the primary accepted method for disseminating scientific results. However, this standard model is no longer the only choice, as its drawbacks and ill consequences have become acutely apparent. First, after completion of the study, the horse is out of the barn—a flawed design or analysis can rarely be remedied post hoc. Additional work to comply with reviewers’ criticism is often biased by the authors’ desire to produce the very results reviewers were asking for. Noncompliance with the reviewers’ “recommendations” often leads to rejection, which almost invariably starts a cascade of submissions, spiraling down the “hierarchy” of journals. Eventually, manuscripts will get published somewhere. It has been argued that post hoc reviewing and the cascade of multiple reviews wastes time and resources of authors and reviewers without substantially improving science and results in an inflation of the publishing record with questionable studies.

In addition, presenting studies after completion and analysis of its results invites selective use of data (“cherry picking”), nonpublication of results that do not fit the hypothesis or even contradict it, hypothesizing after the results are known (HARKing), and storytelling. A telltale sign of such practices is the common style of articles in top tier journals, in which most paragraphs of the result section start with “Next we ….” Studies are presented as a linear trajectory of experimentation, following a logical sequence dictated only by the ingenuity of the researcher and her/his research question or a priori hypothesis. Arguably, these practices, in combination with deficient internal validity (e.g., control of biases) and statistical flaws (e.g., insufficient power) underpin the current reproducibility crisis. Fortunately, all these issues can be resolved in one stroke, at least in principle, through preregistration and Registered Reports [4].

## Clinical research to the rescue—Preregistration

Bugged by similar problems as those currently afflicting basic and preclinical biomedical research as well as psychology, clinical research has already developed and implemented an effective fix: the preregistration of study protocols in a trial registry (such as Clinicaltrials.gov) before the first patient is enrolled. Most clinical journals publish trials only if they were preregistered. It must be noted, however, that clinical trials quite often have only one major endpoint, as well as standardized and auditable methodology that needs to comply with the strict rules of good clinical practice (GCP). The research question can usually be phrased as a straightforward hypothesis. In addition, a lot of knowledge supporting the efficacy and safety of an intervention has already been accumulated before it can be tested in humans. Conceptually, most clinical trials therefore have the characteristics of a confirmation.

Building on the experience from clinical trial protocols, registration of study protocols (e.g., with the Open Science Framework) is gaining substantial traction in some nonclinical fields, in particular, psychology. Protocol registration has been further developed into a structured article format, so-called “Registered Reports,” which are now adopted by many journals, including *PLOS Biology*. A Registered Report is an article in which the methods and proposed analyses are preregistered and peer reviewed prior to the study being conducted. This review process, and potential modifications to the study design based on expert feedback, leads to provisional acceptance of studies that are deemed methodologically sound (stage 1). Once the study is completed, authors submit a full manuscript for final review (stage 2). The editorial decision will not be based on the results of the study but on quality checks and a sensible interpretation of the findings by the authors. Registered Reports thus antagonize the inappropriate research practices mentioned previously, including inadequate statistical power, selective reporting of results, undisclosed analytic flexibility, and publication bias.

## Preregistration for exploratory research?

Preregistration and Registered Reports ask for a prespecification of the hypothesis, primary outcome, and mode of analysis, which makes them ideally suited for studies aiming to confirm previous research results. But a pressing question is whether they can be applied to exploratory research as well. In exploration, researchers are facing a virtually unbounded landscape of potential mechanisms, targets, drugs, doses, and treatments. Exploration develops theories and hypotheses, measurement techniques, and generates evidence that justifies further research or selects a manageable number of interventions to carry forward [5].

Indeed, the current biomedical literature is dominated by exploration discovery of novel mechanisms or treatments. We should not be deceived by fact that discovery often doubles as confirmation: many published articles claim that they have discovered a phenomenon and confirmed it in the same experiment. This is a logical fallacy, as the same data cannot be used to generate and test a hypothesis. Exploratory investigation aims to generate robust theories about biological mechanisms, not to confirm them. Exploration at the frontiers of present knowledge inherently produces a substantial rate of false positive results. Confirmatory research must follow to weed out false positives and correct inflated effect sizes. In discovery mode, researchers probe as-yet-unknown biological mechanisms with a multitude of complex state-of-the-art methods. They may start with an a priori hypothesis and a tentative plan of how to conduct and analyze their experiments. But through serendipity, novel information obtained through scientific communication, ingenuity, etc., they often end up with unexpected results obtained via an unanticipated research strategy. It is quite obvious that this enormous amount of scientific freedom makes exploratory research highly susceptible to the undesirable practices mentioned previously, in particular, uncontrolled bias, low power and flawed statistics, as well as undisclosed selective use of data. Can preregistration and Registered Reports be applied to mitigate such research practices in exploratory studies?

## Charting the unknown waters of biology

There are interesting parallels to the original world of exploration. Masses of uncharted water and land were crossed and mapped, mostly motivated by fame, fortune, and the European nations’ quest for riches. Exploration at the frontiers of modern biology may be mainly driven by human curiosity, but individual and national advancement and commerce are still important motives. Explorers like Magellan or von Humboldt relied on maps and landmarks charted by their precursors but did not know what lay ahead of them. Similarly, researchers today sail through an ocean of ignorance of infinite size, using landmarks of existing knowledge—which they often revise in the process. They triangulate their route using different methods, not compasses and sextants, but combinations of gene targeting, immunohistochemistry, or pharmacological manipulation [6]. On their journey, researchers proceed in an inductively deterministic manner, usually unaware of the many levels of freedom available to them. These arise, for example, from alternative analysis or interpretation of their experiments, false positive or false negative intermediary results, or the multitude of potential methodological approaches. As a consequence, there is not merely one way to cross the ocean of biology but many. And just as Columbus, researchers may end up in America—not, as planned, in Asia—and may nevertheless be convinced that they had reached the coastline of the Spice islands.

But in contrast to today’s researchers, explorers “preregistered” their journeys, usually with the sovereigns financing their expeditions. And more importantly, they mapped their complete travels, including deviations and disasters, sending reports home while still pursuing the unknown. This improved the maps for others following them, making future expeditions safer and more effective. Analogously, researchers could—before setting sail (or rather, sitting at the bench)—specify a destination in the form of a hypothesis or putative mechanism (i.e., specify the core research question) and set up some tentative advance rules according to which experiments and analyses are planned. This could be based on their previous voyages (for example, pilot data) but also on already existing maps, that is, the published literature. Such a plan could be preregistered or be used for stage 1 of a Registered Report. During the voyage, in particular, when novel data are obtained through triangulation with various methods, they could update a log, maintained alongside the registered protocol and later made available to reviewers and readers. This would happen every time new data come in or decisions about how to proceed next are made. Ultimately, the log would chart the entire trajectory of a study, justify the selection (or omission) of data, and even capture failed lines of experimentation.

## Why bother preregistering exploratory research?

What would be gained by preregistering exploratory research and associating the registration file with a log? Stage 1 peer review would alert researchers about methodological and analytical weaknesses, conflicts with guidelines, overlooked or misinterpreted previous evidence, etc. This would help them to increase study quality before they start, potentially saving resources and even animals [7]. Through the log, reviewers could monitor the work as it unfolds, a “rolling review,” as proposed by Chambers and Tzavella [8]. Alternatively, the log could be kept as an open laboratory notebook [9]. The ensuing log would lead to a real “next we” narrative, not the imaginary post hoc storytelling that is presently common. It would expose the meandering of the research process and the many choices available and justify those eventually selected by the scientists during the course of the study. Methodological and analytic flexibility is maintained but disclosed. Outcome-switching might nevertheless occur, but labeled as such and under the eyes of the reviewers and readers, they would lose their stigma. Preregistration combined with logging of study progress preserves the freedom of the explorer and the power of serendipity.

Details of this protocol and log would need to be further specified, but in principle, they are variants of “incremental registrations,” which have already been established by various journals. Surely, researchers could game such a system, for example, by selectively logging experiments. However, they would forgo the benefits of potentially receiving valuable input before the study starts. Even more importantly, a major strength of exploratory research protocols would be that through them, researchers—especially if they feel the urge to be selective about their reporting—become self-aware of the inherent limitations of discovery research. This includes the use of test statistics in exploration, which, according to Gelman and Lokens, can become a “machine for producing and publicizing random patterns [10]." Preregistration and logging would make authors and readers more skeptical and wary of unrealistic conclusions.

## Selectivity: Great science versus rigor and technical soundness?

A major strength of Registered Reports is that they focus the review process on the research question and soundness of methodology. Journal selectivity strongly impacts the rigor, reproducibility, relevance, and transparency of the publication record. Current biomedical journals can be loosely stratified into 2 groups according to the editorial criteria for publishing research (in other words, their selectivity). Many journals—in particular, those at the top end of the hierarchy—emphasize “great science,” “fundamental insights,” “scientific importance,” or “outstanding significance.” On the other end of the spectrum are journals that emphasize rigor and technical soundness over originality or relevance and encourage null and negative findings or reanalyzes.

But isn’t it great science that drives progress? And isn’t great science, by definition, rigorous and technically sound? Does shifting selectivity toward rigorous and relevant investigation and away from judging the novelty of their results lead to boring publications and a contamination of the publication record with null results? Theory, meta-research, and a plethora of highly publicized examples indicate that spectacular results, which promise major advances, unfortunately must often be nonreproducible or even false [11]. Authorships in *Nature* or *Cell* in many countries, including Germany, almost guarantee future funding and tenure. An exclusive focus on work of outstanding significance strongly incentivizes researchers to “produce” such results by sacrificing rigor and sometimes even through misconduct. This can only be remedied by changes of the academic reward system [12]. But these changes can be supported by implementing, popularizing and incentivizing novel article formats that de-emphasize the focus on desired results. Protocol registration and, in particular, Registered Reports are an ideal step in this direction. Tenure committees or grant reviewers should consider valuing a Registered Report in *PLOS Biology* or *F1000Research* just as much as a paper in *Nature*.

As the need for confirmatory studies is becoming more widely accepted, and structured initiatives are spreading (e.g., Brazilian Reproducibility Initiative, Reproducibility Project: Cancer Biology), preregistration and registered protocols will become more common. However, the bulk of scientific investigation in those fields is exploratory. Journals and scientists need to modify the existing preregistration formats and experiment with them. It is plausible, but so far unproven, that emphasizing the relevance of the research question and methodological rigor when selecting studies for publications will reduce waste and increase value of biomedical research. I posit that the reduction of underpowered studies and overblown interpretations, the availability of negative and neutral results for evidence synthesis, the reduction of false positive/false negative studies, as well as greater transparency, will afford the overall reduction in the number of published articles that we are longing for, but those articles that get published will be more trustworthy and useful.

## Abbreviations

GCP: good clinical practice
HARKing: hypothesizing after the results are known
